# Evaluation of the Unit Rod surgical instrumentation in Duchenne scoliosis. A retrospective study

**Published:** 2016

**Authors:** T Nedelcu, I Georgescu

**Affiliations:** * Paediatric Orthopaedics Department, General Hospital, Le Havre, France; ** Paediatric Orthopaedics Department, Paediatric Physical Rehabilitation Centre Bois Larris, Lamorlaye, France

**Keywords:** Muscular dystrophy, Duchenne, scoliosis, vertebral arthrodesis, Unit Rod

## Abstract

The article represents a retrospective clinical and radiological study.

**Objectives.** Evaluating the safety and efficiency of the surgical treatment by using the Unit Rod for scoliosis in adolescents and children presenting Duchenne’s muscular dystrophy.

**Summary.** Surgical management of myopathic scoliosis still causes controversies regarding the timing of surgery (patient’s age), the pelvic inclusion in the arthrodesis or the advantages of surgery over the conservatory treatment. The patients are very fragile and a long surgery with massive blood loss could lead to serious complications. Unit Rod instrumentation is simple, confers excellent stability and has a low rate of complications.

**Methods.** This is a retrospective clinical and radiological study with a medium follow-up of 6.9 years including 13 patients diagnosed with Duchenne myopathy. All investigated patients were non-ambulatory at the time of surgery and have been treated by the Unit Rod technique at the University Hospital of Rouen between 2002 and 2008. Spinal fusion was, in all cases, realized from T2 to pelvis. Galveston technique of pelvic fixation and Luque’s sublaminar wire instrumentation of the spine were used.

**Results.** The results obtained with this treatment and post-surgery complications were analyzed and compared with those from literature. The advantages of this technique consist mostly in a good and stable pelvic fixation, a short interventional time, a minimal blood loss and few complications. Cobb angle correction is similar to that obtained by other surgical procedures.

**Conclusions.** Using the Unit Rod instrumentation of scoliosis in Duchenne’s muscular dystrophy is safe, has excellent outcomes, brings post-surgery improvements, and has minor intra and post-surgery complications. The low cost of this treatment could make it a first choice for medical health systems with financial problems.

## Introduction

Duchenne of Boulogne muscular dystrophy (DMD) is a genetic disease that causes progressive muscle degeneration (Guillaume Duchenne described it in 1861 in his work “Paraplegie hypertrophique de l'enfance de cause cerebrale”). The disease is triggered by an abnormality of the DMD gene (defective gene located on the X chromosome) responsible for the production of a protein (dystrophin) involved in sustaining muscle fiber [1]. The frequency of the disease is 1/ 3500 male births. The disease touches males with an absolute majority, with a few exceptions reported in women if the two genes that code dystrophin are pathological. Females are generally only transmitting the genetic abnormality (65%) [**1**].

Signs of the disease occur in childhood before the age of 5, the muscle degeneration touching proximal muscles rather than the distal ones. Pseudo-hypertrophy of the calf muscles is frequently described as a distinctive sign [**2**].

Walking is lost generally before the age of 13. Without treatment, death occurs frequently before the age of 20. The main cause of mortality is an impairment of the heart muscle (cardiomyopathy). Another frequent cause of death is the respiratory arrest, favored by the diaphragm damage [**2**].

Scoliosis occurs in approximately 95% of the patients with DMD with an onset in late childhood or adolescence. The deformity is of average amplitude, the rule being the progression. This progression is even more rapid when the patient’s walking abilities are lost and the wheelchair becomes a way of day life. At this point, scoliosis is usually accompanied by a reversal of the physiological lumbar lordosis. In time, the evolution of this scoliosis leads to a significant lateral tilt of the trunk and one upper limb is being used just for support [**3**].

The scoliosis pattern in this disease is one typical of a neuromuscular scoliosis: single curve, thoraco-lumbar, often including the pelvis. Thoraco-lumbar kyphosis is often associated. Untreated, scoliosis progresses and the accommodation of the patients in wheelchair is badly tolerated, sometimes impossible [**4**].

Conservative treatment with braces has been tried but is currently not recommended as a long-term solution. Despite the brace, scoliosis progresses, diminishing the respiratory function, which is already impaired in these patients (forced vital capacity drops by approximately 4% per year and an additional 4% at every 10° lost) [**4**,**5**].

**Surgical treatment**

In adolescence, the period of rapid growth emphasizes spinal deformities. Spinal fusion is designed to maintain a straight trunk and to favor the function of the respiratory muscles. The main surgical techniques for arthrodesis used today are spinal instrumentation with screws and hooks (according to the technique described by Cotrel-Dubousset) [**6**] and spinal instrumentation using segmental sublaminar wires, a system described by Luque [**7**]. The two techniques can include the pelvis or stop in L5 [**6**-**8**]. The pelvic extension remains controversial, the instrumentation of the pelvis being a source of complications (bleeding, increased surgery time) [**8**-**12**].

**Fig. 1:**
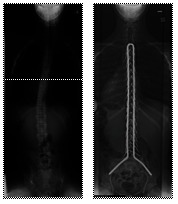
Pre and post-surgery radiographs of a patient with a Duchenne myopathy treated surgically by posterior spinal arthrodesis with the Unit Rod technique. Pelvic obliquity in this case was 0°

Due to the rapid deterioration of the respiratory and cardiac functions after the loss of ambulation, surgery is recommended rapidly once the curve reached 30° or if the patient becomes non-ambulatory. Surgery is easier tolerated before FVC (forced vital capacity) falls below 35% of the normal value for the age [**8**].

## Materials and methods

We retrospectively analyzed the clinical and radiological data of 13 patients with muscular dystrophy of Duchenne de Boulogne presenting a scoliosis, operated by the same surgeon in the Paediatric Orthopaedic Department of the University Hospital of Rouen, France, between 2000 and 2008. The same surgical technique – posterior fusion by using the Unit Rod instrumentation from T2 to the pelvis – was applied for all the patients. Pelvis was instrumented in all the cases without taking into account the pelvic obliquity, but this value was nevertheless recorded in the patients’ files. Follow-up was done at regular intervals post-surgery, with an average time of 6.9 years (the last control being made at the beginning of this study, in 2014) recording clinical data and using X-ray control. All the patients evaluated were male (**Table 1** shows the demographics of patients included in the study).

The inclusion criteria for the study were (for all 13 patients): Duchenne muscular dystrophy diagnosed through muscle biopsy, scoliosis diagnosed clinically and radiologically, non-ambulatory patients. The same surgical technique was used and performed by the same surgeon in the Department of Paediatric Orthopaedics in Rouen, the follow-up being of at least 4 years.

The surgical indication was determined by evaluating clinical factors such as age, immobilization in a wheelchair, imbalance of the trunk in the frontal plane, respiratory and cardiac status and radiological ones like type and progressivity of the curve.

The Unit Rod surgical technique was used for all the patients, this being the technique preferentially used for neuromuscular scoliosis surgery in our Department. The surgical correction consisted in a posterior spine fusion from T2 to the pelvis by using sublaminar wires at every level and the Unit Rod, which was inserted in the pelvis by using the Galveston procedure [**7**]. The wires were then passed around the rod, tightened and though the curve being corrected.

**Table 1:** Patients’ demographics

Name	Sex	Birthdate	Walker/Non-Walker W/NW	Year of the intervention	Aga at the surgery (years+months)	Follow up (years)
B.J.	M	28/08/1989	NW	2004	15+0	10
B.V.	M	15/11/1994	NW	2008	13+4	5
B.B.	M	23/05/1992	NW	2004	11+11	5
D.Q.	M	06/10/1993	NW	2005	11+7	8
F.G.	M	16/10/1991	NW	2006	15+0	8
F.R.	M	02/08/1988	NW	2002	13+4	6
G.N.	M	22/10/1990	NW	2002	11+7	4
H.B.	M	20/04/1990	NW	2003	12+10	5
L.L.	M	04/06/1997	NW	2008	11+2	6
L.J.	M	13/07/1993	NW	2006	12+10	7
M.A.	M	20/07/1992	NW	2003	11+3	9
M.F.	M	15/06/1988	NW	2002	13+9	10
R.V.	M	07/08/1989	NW	2002	12+9	7

No graft from the iliac wing was needed, only the spinal and transverse processes were used to assure the fusion.

The Unit Rod instrumentation proved to be more effective than the use of two independent rods in preventing the crankshaft effect [**13**].

The major coronal curve was measured on A-P radiographs by Cobb’s method. Pelvic obliquity was calculated on the same X-ray as the angle between the tangent line at the two iliac crests and a horizontal line.

**Fig. 2:**
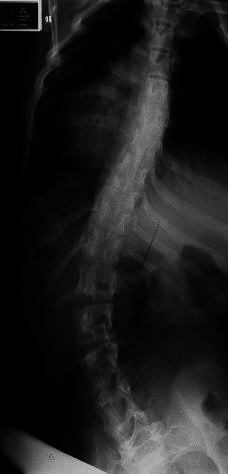
Pre and postoperative radiographs of a patient with Duchenne myopathy treated by posterior spinal arthrodesis using the Unit Rod. In this case, the pelvic obliquity was of 30° before the surgery and was reduced to 0° postoperatively

## Results

The average follow-up was of 6.9 years with a range between 4 and 10 years. All 13 patients included in the study met all the criteria with a comprehensive medical record and preoperative, immediate postoperative and at the last follow-up the existing X-rays. The mean age of patients at the date of surgery was 12.7 years (11-15 years). The mean age was lower than in other patients with neurological diseases, being conditioned by the respiratory and cardiac functions of these patients.

Postoperative outcomes assessment took into account the length of the surgery, the intra-operative bleeding, the intra and postoperative complications, the radiographic dynamics and the patient’s clinical status.

**Radiological results**

Cobb angle correction was of 94% immediately post-surgery and of 87% at the last check (more than 4 years past surgery). This extensive correction was possible because patients with myopathic scoliosis were operated quickly, when the Cobb angle had small values and the scoliotic curve was still very flexible. The bending radiographs confirmed this spinal flexibility pre-operatively (**Table 2**).

**Table 2:** Dynamics in radiological parameters

Radiological Parameters Pre-operatively		Post-operatively	
		Immediately > 4 years	
Cobb angle (°)	30 (25-40)	2 (0-10)	4.15 (0-12)
Pelvic obliquity (°)	7.7 (0-15)	0.6 (0-5)	1.5 (0-10)

**The correction of pelvic obliquity**

It was demonstrated that although most patients with this pathology showed a small obliquity of the pelvis, below 15°, it could aggravate if the pelvis was not included in the fusion [**10**]. Our results showed a 95% correction of the pelvic obliquity immediately after the operation and an 85% correction at last follow-up. These figures remained constant in time for all the patients, with the exception of one case, in which the pelvic insertion of the rods created a lysis chamber, leading to a progressive loss of the correction even though the pelvic obliquity increased by 10° (**Table 2**).

**Clinical outcomes**

Improvement in the clinical status of the patients [**22**,**23**] after surgery was assessed in the current study through a satisfaction survey questionnaire SRS (Scoliosis Research Society) modified in our clinic for children carrying a Duchenne dystrophy. Although this form was not validated, it is in our opinion suited for the evaluation of these patients.

**Fig. 3:**
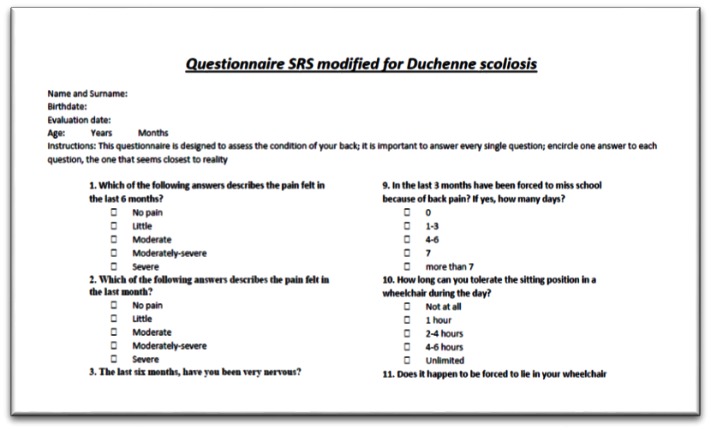
Questionnaire assessing clinical outcomes after surgery

The questionnaire assessed: the pain (in the wheelchair or lying down), the tolerance of the wheelchair, the change in the quality of life once without brace, the ischial pressure sores frequency, the psychological status and the overall satisfaction.

Patients described occasional pain (with one exception – a patient who presented medium-high pain on a regular base), an improvement in the comfort of life once without brace, decreased frequency of pressure sores (probably due to the improved balance of the pelvis), a very good mental status, good tolerance of electric wheelchairs and quality social integration.

**Fig. 4:**
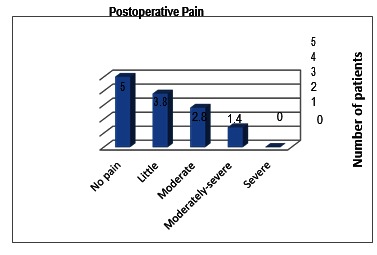
Number of patients who have experienced various degrees of pain in the past 6 months

**Fig. 5:**
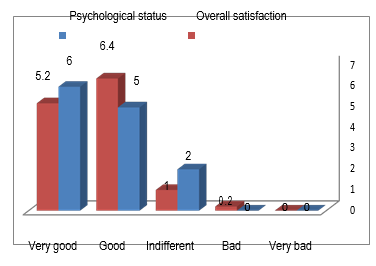
Distribution of patients depending on overall satisfaction and psychological state at last follow-up

**Fig. 6:**
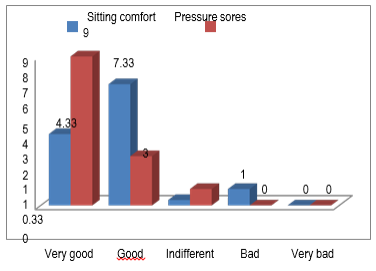
Distribution of patients depending on the wheelchair tolerance and pressure sores frequency.

**Operative time**

Operative time is known as an important factor in limiting postoperative infectious complications. In our study, the mean surgery time was of 170 minutes (range between 120-210 minutes), lower than for the same interventions when pedicle screw fixation was used (this technique takes a mean time of 269 minutes for a single posterior approach and of 340 minutes for anterior and posterior approach in our Department in Rouen).

**Intra-operative bleeding**

Estimated intra-operative bleeding (EBL – “Estimated blood loss”) was recorded in the anesthetics records during the operation and represented the only practical way of assessing the intra-operative blood loss [**14**].

It remains imprecise being calculated as a sum of the volume of blood suctioned from the operating field (minus the volume of liquid irrigation), the determined blood loss collected on sponges (as determined by weighing the sponges), and estimated blood loss on drapes and floor [**14**].

It is recognized that patients with neuromuscular diseases show a higher risk of bleeding than patients with idiopathic scoliosis - up to 7 times higher according to some studies [**3**]. For the patients with muscular dystrophies, the lack of dystrophin further increases intra-operative bleeding [**1**].

In the present study, the mean blood loss was estimated at 2553.8 ml with ranges between 1800 ml and 3500 ml, representing 74.2% of the estimated blood volume (EBV), ranging between 58.4% and 96%. Patients who required transfusion were the ones suffering blood losses of more than 74% of the EBV (**Table 3**).

**Table 3:** Estimated intra-operative blood loss

Name	Age at the intervention (years)	Weight at the operation	Estimated blood volume Pre-operatively	Estimated blood loss EBL (ml)	% of the blood volume lost intra-operatively	Patient transfused intraoperatively
BJ	15	52	3640	3000	82.4	Yes
BV	13	47	3290	2200	66.8	No
BB	12	37	2590	2000	77.2	Yes
DQ	11	57	3990	2500	62.6	No
FG	15	65	4550	2600	57.1	No
FR	13	46	3220	2900	90	Yes
GN	11	35	2450	2200	89.8	Yes
HB	12	44	3080	1800	58.4	No
LL	14	63	4410	2800	63.5	No
LJ	12	45	3150	2000	63.5	No
MA	11	50	3500	2600	74.2	Yes
MF	13	52	3640	3500	96.1	Yes
RV	13	53	3710	3100	83.5	Yes
Mean				2554	74.2	53.80%

The estimated blood volume in patients was calculated according to the formula: 70ml/ kg x patient’s weight in kg, as described in Shapiro’s study [**14**].

This bleeding is comparable to that cited in literature for DMD – 2500 ml to 4000 ml [**14**]. It seems it depends on the number of instrumented segments, the surgical time, and the adopted surgical technique. Iliac graft and pelvic inclusion in the fusion are also associated with an increased intra-operative bleeding. In the present study, arthrodesis being performed systematically from T2 to pelvis, the number of the instrumented vertebrae was not an element impacting the blood loss.

7 of the 13 patients required intra-operative blood transfusions (53.8%). These transfusions were decided by the anesthetist in accordance with the intra-operative decrease in the haemoglobin level under the limit allowed by the department’s protocol (transfusion is considered necessary intra-operatively when Hb ≤ 9 g/ l and post-operatively when Hb ≤ 8g/ l). It was recorded in the surgical file that only two of these patients presented an excessive intra-operative bleeding.

## Complications

Complications were minimal in intra-operative time and without significant consequences in post-operative period. They were represented by: a false path in the iliac crest when placing the stem of the Unit Rod in the pelvis; it was corrected by changing the rod inclination and creating a new path in the iliac wing (1 patient); intra- operative abundant bleeding (2 patients); rupture of the posterior arch of L5 (1 patient).

Postoperative complications were represented by: difficulties in scarring/ wound disunion (3 patients); site infections (2 patients) - these patients had a surgical re-intervention with a surgical lavage, local debridement and antibiotic treatment according to our department’s protocol and did not require the removal of osteosynthesis material; sublaminar wire breakage in T2 (1 patient) without secondary loss of correction: the rod was visible under the skin without impingement and there was no need to re-operate; lysis around the intra-pelvic insertion of the rod of more than 2 mm with secondary loss of correction of pelvic obliquity - 10° loss (1 patient).

## Discussions

Duchenne scoliosis treatment is complex and well staged today.

Corticosteroid therapy started before the loss of ambulation and respiratory degradation often delays the moment of appearance of the spinal deformity. However, the onset of scoliosis is inevitable in the evolution of this pathology [**2**].

Conservative treatment (bracing), by allowing a comfortable sitting position, is a temporary solution increasing the waiting time for the surgical decision. Although this type of spinal fusion is practiced earlier than in other neuromuscular scoliosis (12.7 years for Duchenne and 15 years for other neuromuscular scoliosis in patients treated in the Paediatric Orthopaedics department in Rouen) due to respiratory and cardiac problems, sometimes surgery may be delayed by a number of factors that cannot always be met simultaneously: consistent clinical follow-up, difficulties in scheduling the operation, availability of family and patient for surgery, etc.

Spinal fusion remains the treatment of choice even if sometimes delayed. For the DMD patients the approach is always a unique posterior one, the anterior approach not being necessary in view of the small magnitude and the flexibility of the curves [**15**]. The choice of fixation material depends on many factors: the existing protocol in the department, the surgeon’s experience, the cost of the material and the associated pathologies of the patient.

Currently, there are two main techniques used for these patients: segmental instrumentation with screws and hooks according the Cotrel-Dubousset principles and segmental techniques with sublaminar wires according to Luque’s principles, with or without pelvic fixation.

This type of surgery, regardless of the technique used, remains difficult, grafted by multiple complications, intra-operative significant bleeding and requires a specialized pre, intra and post-operative environment equipped for this type of intervention. Some teams recommend an additional period of postoperative assisted ventilation (days or weeks) in order to speed up the recovery of the vital capacity after the intervention.

The Unit Rod technique for spinal fusion is commonly used in the treatment of neuromuscular scoliosis. [**7**,**8**,**13**]. There are many papers in literature showing the advantages of this technique [**16**-**21**]. One of the indications for this technique is the non-ambulatory patient with a scoliosis presenting the Duchenne myopathy. The patient being in a wheelchair at the time of the operation and inclusion of the pelvis in the fusion does not have a negative impact by changing the sacral slope and blocking the lumbosacral mobility. The approach is always unique, posterior, with segmental instrumentation using sublaminar wires. The Unit Rod is inserted through the two distal stems in the iliac wing, according to the Galveston technique [**7**].

The extension of the fusion to the pelvis is provided by this system, but also done in many patients treated with pedicle screws segmental techniques, but remains a controversy [**6**,**8**-**12**]. Some authors like Mubarak al. [**8**] believe that if there is no pelvic tilt at the time of the surgery, instrumentation should stop in L5. Alman and Kim [**10**] observed a pelvic obliquity progression, if instrumentation stopped in L5, in about 30% of the patients operated, even those with minor or no pelvic obliquity at the moment of the fusion. Therefore, their recommendation is pelvic fusion systematically.

Patients with neuromuscular scoliosis have an increased rate of intra-operative bleeding (up to 7 times greater than in the case of idiopathic scoliosis). Sublaminar wire instrumentation for spinal fusion in DMD is described by Arun et al. [**3**] as one of the bleeding technique (compared to a hybrid technique or pedicle screw technique). In their study, using the Luque–Galveston technique blood loss amounted up to 4000 ml and the pedicle screw technique had the lowest rate of blood loss of approximately 2500 ml. Nevertheless, the two groups of patients were not perfectly matching: the DMD scoliosis patients operated by the Luque technique showed a mean Cobb angle of 50° while for the hybrid and pedicle screws techniques the mean Cobb angle was 25° and 17°. For the patients operated through the last two methods, the pelvis was never included in the arthrodesis.

Our study showed blood loss similar to those found by Arun et al. for pedicle screws technique. It was assumed that if pedicle screws were used to extend the fusion to the pelvis [**24**], the blood loss would have increased, exceeding the losses described in our study.

The study by Shapiro et al. [**14**] looks through a review of several studies regarding blood loss for DMD patients operated by the Luque-Galveston technique ranging between 2500 to 4000ml.

The rate of complications for these interventions is very high (approx. 30% in the literature). Complications commonly described in patients with Duchenne myopathy are: important bleeding, postoperative infections, the destabilizing of the instrumentation and lung infections [**3**,**24**]. The Unit Rod is a technique faster than pedicle screw instrumentation through (170 minutes versus 210 minutes in our department in Rouen), thereby decreasing the risk of bleeding, transfusion rate and infectious complications.

Spinal stabilization does not seem to increase life expectancy for patients with Duchenne myopathy, but significantly improves the quality of life and reduces the rate of decline in FVC.

## Conclusion

Posterior spinal arthrodesis remains the intervention of choice for most cifoescoliosis in myopathic scoliosis. The timing of the intervention is important: being ideal after the period of rapid growth (after closing of the tri-radiate cartilage to avoid the crankshaft effect), but not much later in order to avoid the risk of cardiac and respiratory degradation in these patients, a higher Cobb angle and a lack of flexibility of the spine, which leads to an increase of risks.

In most cases, arthrodesis allows the non-use of a brace, improves the installation of the patient in a wheelchair and the quality of life in these patients.

The type of technique used in the present study (Unit Rod) is proved to be a simple, cheap and effective treatment of scoliosis in patients with Duchenne muscular dystrophy, representing an ideal choice in any department of paediatric orthopaedic surgery. It is the perfect instrumentation for medical health systems with financial problems.

Intra- and post-operative complications are rare, equivalent to those found in other procedures, our study showing the functional benefit obtained in most operated patients.
